# Evaluation of a Consumer Wearable Smartwatch for Cardiac Interval Measurement in Healthy Adults: Cross-Sectional Method Comparison Study

**DOI:** 10.2196/90009

**Published:** 2026-09-04

**Authors:** Nicholas Noggle, David Wing, Job Godino, Michael Higgins, Daniel Moreno, Dylan Lawton, Ariel Dowling, Jasmin Imsirovic, Ryan Moran

**Affiliations:** 1 School of Medicine, University of California San Diego, La Jolla, CA, United States; 2 School of Medicine, Albany Medical College, Albany, NY, United States; 3 Herbert Wertheim School of Public Health and Human Longevity, University of California San Diego, La Jolla, CA, United States; 4 Exercise and Physical Activity Resource Center, University of California San Diego, 9500 Gilman Drive, dept #0811, La Jolla, CA, 92093, United States, 1 (858) 534-9315; 5 School of Medicine, University of Hawaii Cancer Center, Honolulu, HI, United States; 6 Digital Health Sciences, Takeda (United States), Cambridge, MA, United States

**Keywords:** arrhythmia, cardiac, wearable devices, wearables, electrocardiogram, ECG, RR interval, QT interval, corrected QT interval, QTc interval

## Abstract

**Background:**

Commercially available wearable devices, capable of measuring cardiac rhythm, are gaining popularity for convenient heart health monitoring. Accurate measurement of key intervals, specifically RR intervals for rhythm and QT and corrected QT (QTc) intervals for arrhythmia risk, is crucial for assessing potential cardiac morbidity.

**Objective:**

This study aimed to evaluate the agreement of RR, QT, and QTc intervals measured by a consumer wearable device (Withings ScanWatch series 1) with those measured using a research-grade 3-lead electrocardiogram (ECG; Biopac) and a clinically approved single-lead patch (Cardea SOLO).

**Methods:**

This was a cross-sectional study of 28 healthy adults aged 21 to 65 years, with a mean age of 39.4 (SD 13.0) years. Participants underwent 4 concurrent ECG measurements per device: twice seated at rest, once supine, and once after exercise. All RR and QT intervals were manually measured using standardized caliper software (EP calipers) by trained reviewers, and QTc was calculated using the Bazett formula. Agreement was assessed via repeated-measures correlation (*r*_rm_) and linear mixed-effects models (LMMs) for repeated-measures Bland-Altman analysis.

**Results:**

Strong agreement was observed for RR intervals when comparing the Withings ScanWatch with both the Biopac (*r*_rm_=0.84; *P<*.001) and the SOLO (*r*_rm_=0.80; *P*<.001). Agreement was weaker for QT intervals (*r*_rm_=0.37; *P*=.001 compared to Biopac and *r*_rm_=0.37; *P*=.001 compared to SOLO) and QTc intervals (*r*_rm_=0.27; *P*=.01 compared to Biopac and *r*_rm_=0.27; *P*=.03 compared to SOLO). Bland-Altman LMM analysis demonstrated that the Withings ScanWatch systematically underestimated QT intervals, with a mean error (ME) of −42.9 (SD 30.1) milliseconds versus Biopac and −15.8 (SD 20.6) milliseconds versus SOLO. Similarly, QTc intervals were underestimated, with an ME of −51.4 (SD 32.3) milliseconds versus Biopac and −18.8 (SD 21.5) milliseconds versus SOLO.

**Conclusions:**

While the Withings ScanWatch showed strong agreement for RR interval measurement, its variability and systematic underestimation in QT and QTc measurements limited its clinical applicability for accurate assessment of arrhythmia risk. The device may serve as a valuable tool for tracking trends over time, but it is currently unreliable for precise interval screening.

## Introduction

Arrhythmias are common and have serious clinical and public health implications, imposing a substantial health care burden. While atrial fibrillation (AF) is the most prevalent clinically significant arrhythmia [1,2], other less frequent arrhythmias that present with irregular or elongated intervals within portions of an electrocardiogram (ECG) tracing are often detected in ambulatory settings [3,4]. Monitoring metrics such as the RR interval (for heartbeat regularity) and the corrected QT (QTc) interval (for repolarization abnormalities) are crucial, as elongations can indicate life-threatening events such as *torsade de pointes* [4,5]. Effective, accessible screening is therefore essential for guiding clinical decision-making [6,7].

Traditionally, clinicians assess arrhythmias using in-clinic 12-lead ECGs, Holter monitors, or event monitors. Although the 12-lead ECG remains the diagnostic standard, constraints such as relative inaccessibility and the requirement for physician presence limit its utility for instantaneous, frequent measurement [8]. Ambulatory options, such as 48-hour Holter monitors or single-lead event patches (eg, Cardea SOLO), offer extended tracking but remain cumbersome and are typically only prescribed after a patient experiences intermittent symptoms [9-12]. Consequently, asymptomatic or transient arrhythmias often go undetected. For example, a study by Turakhia et al [13] estimated that in 2009, AF affected 5.3 million people in the United States, with 700,000 cases remaining undiagnosed.

In recent years, consumer wrist-worn devices capable of episodic, user-initiated ECG acquisition have emerged as a potential solution for patient-centered, “just-in-time” monitoring. The increasing ubiquity and convenience of these devices could facilitate early detection of transient arrhythmias well before clinical symptoms arise, potentially streamlining population-level screening [14,15]. While devices such as the Apple Watch have demonstrated promising precision for QT and QTc interval measurement compared with 12-lead ECGs [8,16], other commercially available smartwatches remain largely unvalidated.

One such unstudied device is the Withings ScanWatch. Unlike predominantly digital wearables, the ScanWatch was designed as a hybrid analog timepiece specifically targeting consumers who prefer traditional analog watch aesthetics. Crucially, it was the first hybrid smartwatch to achieve Food and Drug Administration (FDA) clearance for user-activated single-lead ECG measurement [17]. Given its unique market position and higher-risk target user base, establishing its independent diagnostic precision against clinical reference standards is important. Specifically, there is a paucity of evidence comparing consumer-grade wearables with traditional ambulatory monitors for the precise measurement of RR, QT, and QTc intervals in naturalistic settings.

The current study aims to compare the agreement of beat-to-beat intervals (RR, QT, and QTc) measured by a commercial consumer smartwatch with those measured using both a clinically approved ambulatory monitor (Cardea SOLO, Cardiac Insight Inc) and a high-resolution reference 3-lead wired ECG device (BioNomadix RSPEC 4.3 and BioNomadix Logger, Biopac Systems Inc). By evaluating these devices across distinct physiological states, this study seeks to better understand the diagnostic viability of commercial wearables for continuous heart monitoring with a focus on interval detection.

## Methods

This observational evaluation included 28 participants aged between 21 and 65 years. Participants were excluded from the study if they were unable to walk unassisted; had a diagnosis of cardiovascular, pulmonary, metabolic, and/or autoimmune disease; or had symptoms suggestive of cardiovascular disease, for example, chest pain at rest or with exercise or extreme shortness of breath at low levels of exercise (<5 metabolic equivalents of task [METs]).

### Study Setting and Recruitment

Participants were recruited using physical flyers and existing digital institutional listservs containing individuals from previous studies who had explicitly consented to be contacted for future research opportunities. All concurrent physiological testing sessions were conducted at the University of California, San Diego Exercise and Physical Activity Center across various specialized laboratory rooms optimized for resting and exercise protocols. Data collection occurred between November 2021 and February 2022. The target sample size of 28 participants was established as a convenience sample for a pilot evaluation primarily focused on at-home usability, acceptability, and baseline laboratory validation of metrics. This cohort size aligns with established methodological guidelines for initial clinical device pilot studies, which recommend samples of 20 to 25 individuals to adequately assess instrument reliability and system feasibility prior to large-scale epidemiological deployment [18].

### Ethical Considerations

The study protocol and documents were approved by the institutional review board at the University of California, San Diego (800873). All participants in this study provided written informed consent prior to participating. To safeguard participant privacy and confidentiality, all collected data were managed within REDCap (Vanderbilt University), a secure, password-protected, and limited-access data management platform. Identifying information and deidentified analytical data were stored in completely separate databases within the platform. Cross-database access was strictly restricted to the principal investigator and project manager, who remained blinded to the data until active collection was complete. Participants received US $65 for completing the in-laboratory task and measurement portion of the project, with additional compensation of up to US $130 available for completing subsequent at-home and interview components.

### Devices Used

Upon completion of the informed consent process, participants were fitted with experimental and control devices as follows.

#### Withings ScanWatch Series 1

**Figure 1.**
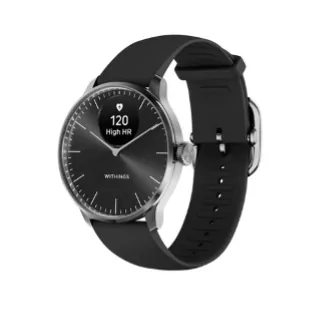
Withings ScanWatch series 1. The watch was placed on the participant’s left wrist following manufacturer recommendations.

This experimental device was placed on the participant’s left wrist following manufacturer recommendations. The watch was put in ECG mode, and the participant started the ECG measurement by pressing and holding the crown, in line with manufacturer instructions. Triggering an ECG measurement engaged the device for a 30-second single-lead ECG tracing. While the device contains an optical photoplethysmography sensor for passive background heart rate tracking, its user-initiated ECG function relies on integrated physical electrodes (the back crystal and outer bezel) to record true electrical cardiac activity when a circuit is completed across both hands (Figure 1).

#### Cardea SOLO

Participants’ chests were shaved as needed for the application of the SOLO patch. The skin on the left side of the chest was cleaned in preparation. The right side of the electrode was placed at the third or fourth intercostal space, close to the sternum. The device was preferentially placed horizontally but was adjusted diagonally as needed based on anatomical constraints.

#### Biopac 3-Lead ECG

The device was applied to participants using standard electrode placement, with a right arm lead placed inferior to the right clavicle, a left arm lead placed inferior to the left clavicle, and a left leg lead placed on the lower left quadrant of the abdomen superior and lateral to the umbilicus. This configuration established a continuous, physical Lead II vector, which aligned closely with the heart’s dominant electrical depolarization axis, making it particularly valuable for the assessment of rhythm-based metrics, including manual QT interval estimation. While a standard clinical 12-lead ECG array provides broader spatial multivector visualization, the 3-lead laboratory reference system was specifically selected for this protocol due to its high-resolution sampling frequency (1000 Hz), continuous data-logging capability, and relative ease of data collection across multiple geographic locations. These technical attributes were strictly required to achieve millisecond-level temporal alignment of corresponding QRS complexes with the episodic, user-initiated 30-second smartwatch triggers. This was critical to establish baseline synchronization.

Both the Biopac and SOLO patch gathered ECG tracings continuously. The Withings-based ECG was gathered 4 times: twice seated at rest, once supine at rest, and once immediately after walking or jogging on a treadmill. Measurements from all 3 devices were taken concurrently during each session. Once the technicians were prepared to collect ECGs, the Withings ECG was triggered, while the other 2 devices continued their continuous measurements. Timestamps marking the start of the Withings ECG were recorded, enabling precise alignment of QRS complexes across all devices.

### Triggering of the ECG

The Withings ScanWatch was placed in “ECG” mode, and the participant was given a countdown to activate the “ECG” function on the watch. The researcher then noted the exact time (hh:mm:ss) of measurement on the Cardea SOLO patch and the Biopac at the beginning of the Withings measurement period. This ensured that temporally identical QRS complexes were analyzed on each ECG produced. The Withings measurement was collected for 30 seconds.

### ECG Acquisition

#### Sitting Down

Participants sat upright in a chair with their arms resting on the table in front of them. ECGs were collected during normal breathing with the participant instructed to sit still with their feet flat on the floor and not speak. Two measurements were gathered with approximately 1 minute between them. These measurements were called trigger 1 and trigger 2, respectively.

#### Lying Down

Participants were instructed to lie down and breathe normally. Once participants were lying flat and breathing normally, the ECG measurement was triggered on the Withings device by a member of the research team. This measurement time point was called supine respiration.

#### Posttreadmill Measurement

In an effort to induce higher heart rates and accelerated breathing, participants walked and jogged on a treadmill at 3 speeds that broadly represented the physical activity intensity thresholds of light (<3 MET), moderate (3 to 6 MET), and vigorous (>6 MET) for approximately 3 minutes at each intensity level. Immediately following this exercise, the participant was seated in a chair and asked to sit quietly, breathing as normally as possible and not speaking. As soon as possible after sitting down, the ECG measurement was triggered on the Withings device by a member of the study staff.

### Measuring RR, QT, and QTc Intervals

A standardized operating procedure for identifying clear ECG signals and placing start and end lines for each interval was developed by an exercise physiologist with extensive experience working with ECG, in consultation with a licensed physician.

The assessment of the individual cardiac tracings was completed by a single researcher with a clinical background who used a standard operating procedure. ECGs collected from each device (Withings, Cardea SOLO, and Biopac) were analyzed using the software program “EP calipers” (EP Studios). Crucially, no automated interval measurements provided by the devices’ internal software or associated applications were used for analysis. The electronic calipers were calibrated prior to each assessment following manufacturer recommendations. Manual measurements of RR, QT, and QTc intervals were gathered on 3 consecutive heartbeats. The analyzed beats were chosen by the reviewer, who selected the clearest ECG tracing from the ScanWatch-based tracing for each condition described above.

**Figure 2.**
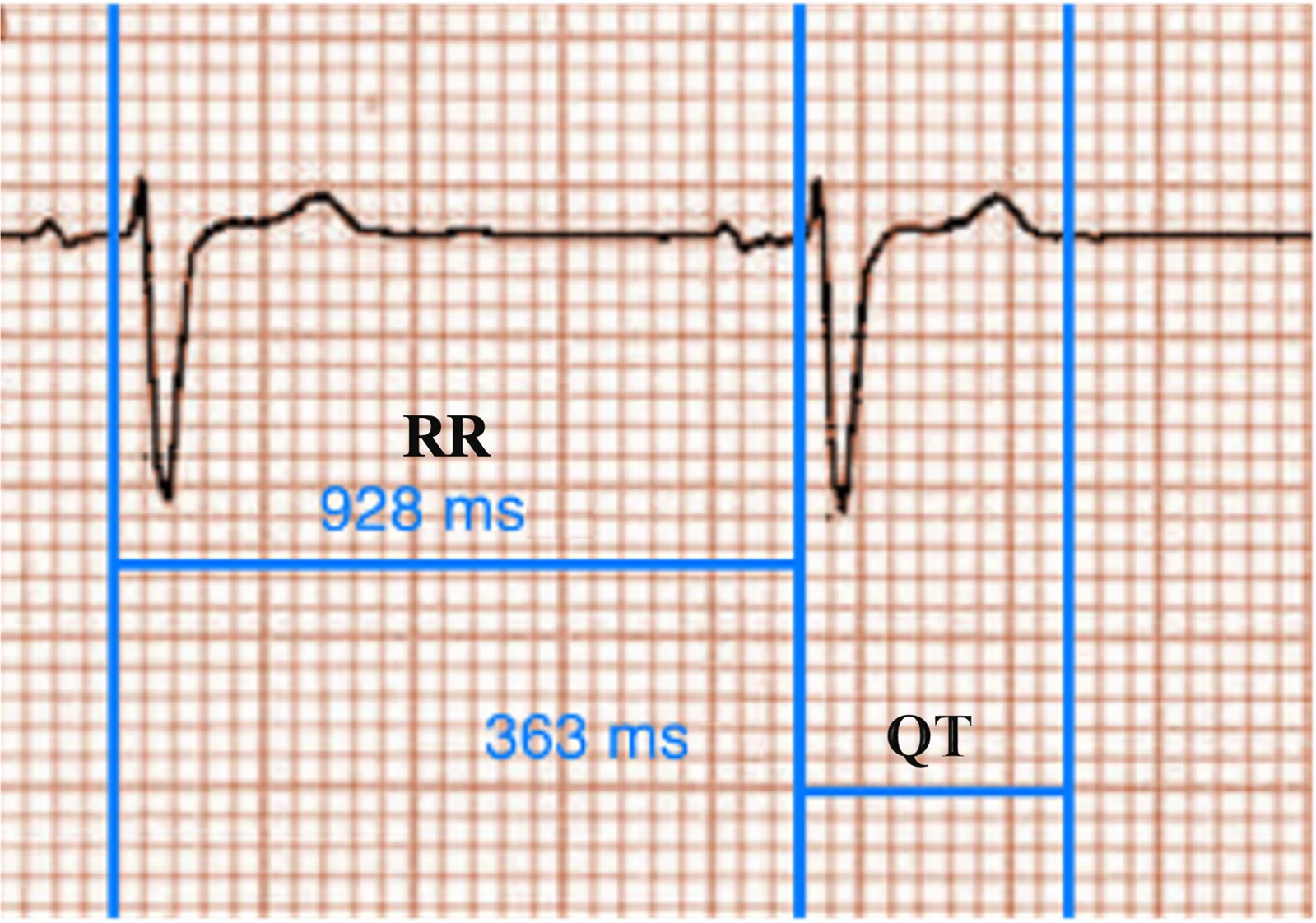
Example of RR and QT intervals measured using EP calipers.

RR intervals were measured by placing the start line of the caliper at the beginning of the Q wave in the first QRS complex and the closing line at the start of the Q wave of the fourth QRS complex. The program then averaged the RR interval over those 3 complexes. The QT interval was measured by placing the start position at the initiation of the first Q wave and the completion line at the end of the first T wave after it had returned to the baseline amplitude. Identical procedures and manual caliper measurements were followed for both the Biopac (3-lead) and Cardea SOLO (1-lead) devices using the same temporal ECG complexes identified by the Withings ScanWatch trigger. The program used the Bazett formula to calculate QTc once RR and QT were recorded. An example of the measurements taken in EP calipers is shown in Figure 2.

### Statistical Analysis

Statistical analysis was performed using SPSS (version 26; IBM Corp). To assess error in the calculation of the intervals of interest, the mean error (ME), mean absolute error (MAE), and mean absolute percent error (MAPE) were calculated as the difference between the Withings ScanWatch measurement and the reference devices. MAPE does not have a standardized threshold for determining the accuracy or validity of measurements. Fokkema et al [19] suggest a MAPE threshold of ≤5%, whereas Nelson et al [20] used a MAPE threshold of ≤10% to classify a wearable device as valid. Studies that used the 5% cutoff when evaluating activity monitors were tracking step count, for which a difference of 5% or greater in step count was considered practically significant [19]. Given the relative difficulty of precisely identifying the end of the T wave, 10% was considered more appropriate than the 5% used by Fokkema et al [19] when considering variables with a relatively easy-to-identify signal, such as steps.

To evaluate interdevice agreement while accounting for repeated measurements within participants across the 4 experimental conditions, repeated-measures Bland-Altman analyses were conducted using linear mixed-effects models (LMMs) with a random intercept for participant ID [21,22]. Fixed intercepts from the models were used to determine the ME and associated *P* values, replacing standard paired *t* tests (2-tailed) to account for within-participant clustering. The 95% limits of agreement (LoA) were calculated by taking the ME plus or minus 1.96 times the total SD, where total SD was derived from the square root of the combined between-participant and within-participant residual variances [21,22]. To assess intraindividual trajectory association across repeated physiological states, repeated-measures correlation (*r*_rm_) analysis was performed. Correlation values were interpreted as follows: 0.10 to 0.39=weak; 0.40 to 0.69=moderate; 0.70 to 0.89=strong; 0.90 to 1.00=very strong.

## Results

### Overview

General characteristics of the participants are presented in Table 1 as means and SDs.

**Table 1.** Participant demographics (N=28).

Sample characteristics	Value, mean (SD)
Age (years)	39.4 (13.0)
Weight (kg)	68.9 (15.9)
Height (cm)	169 (10.1)
BMI (kg/m^2^)	24.2 (5.2)

### Withings ScanWatch Compared With Biopac

**Figure 3.**
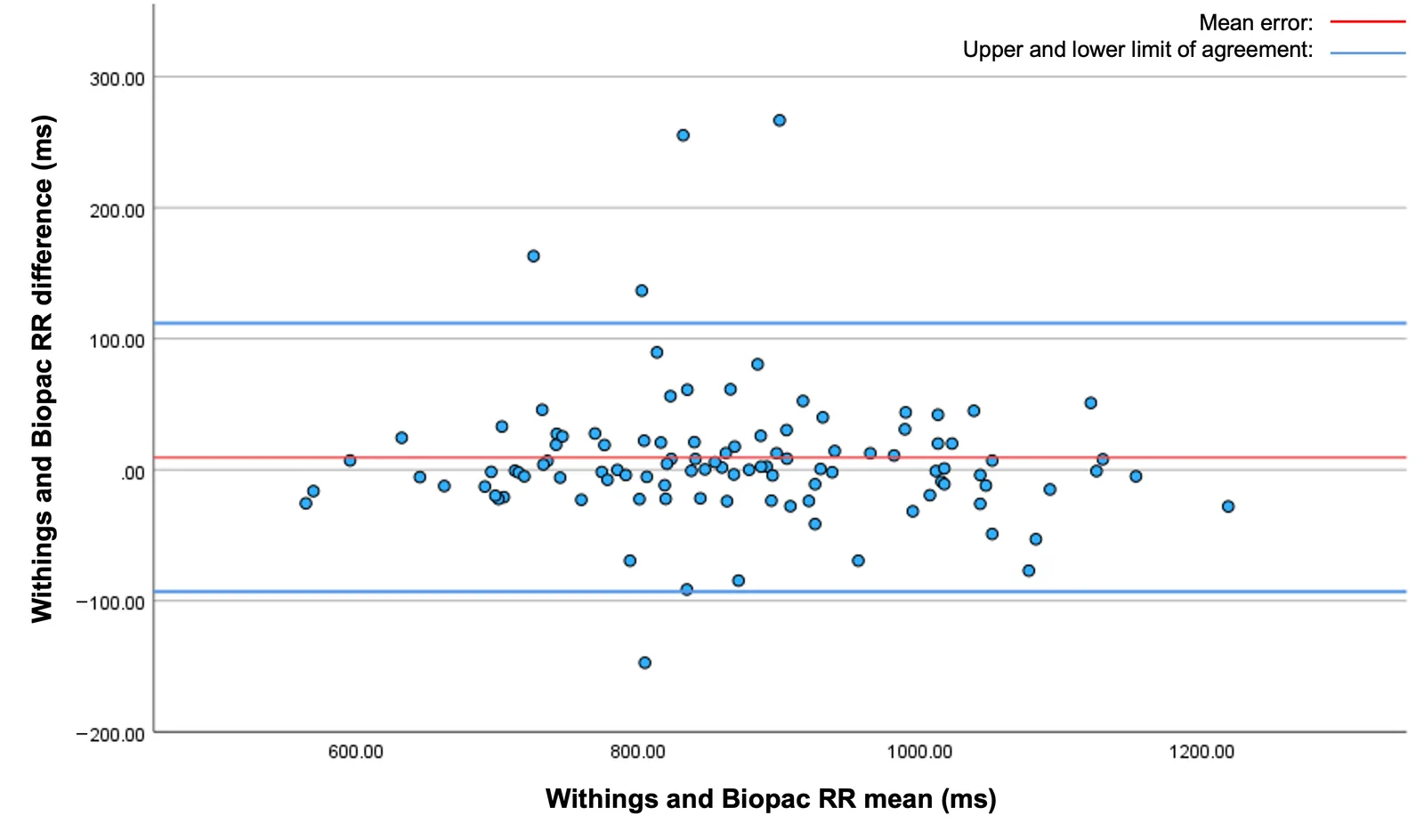
Bland-Altman plot illustrating the measurement agreement of beat-to-beat RR intervals between the Withings ScanWatch and the Biopac laboratory reference system. The plot displays aggregate data points derived from 28 participants evaluated across 4 distinct measurement conditions (seated rest trigger 1, seated rest trigger 2, supine respiration, and posttreadmill recovery). The solid red line represents the mean error, while the upper and lower solid blue lines define the 95% limits of agreement, which were calculated using a linear mixed-effects model to account for repeated measures.

**Figure 4.**
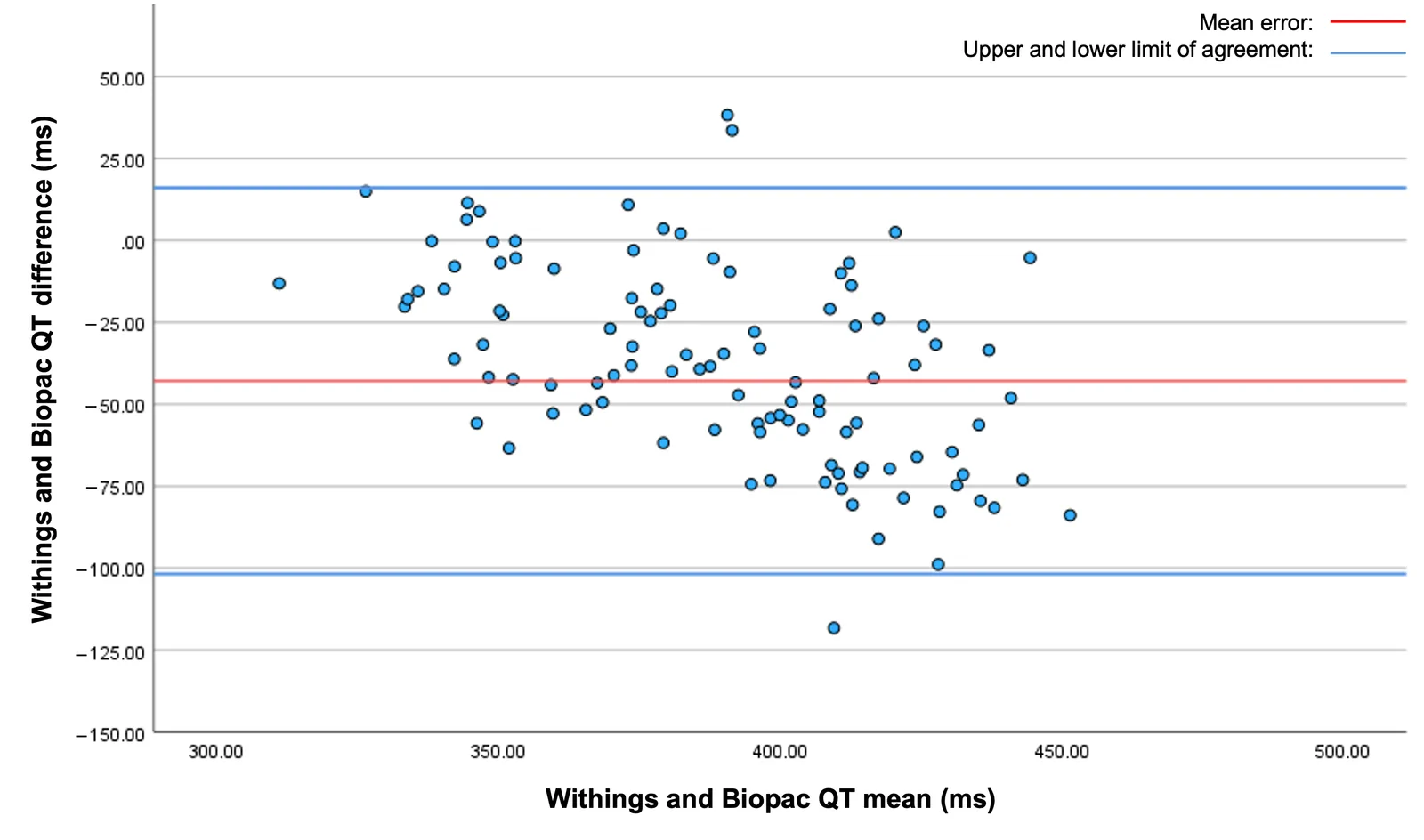
Bland-Altman plot illustrating the measurement agreement of manual QT intervals between the Withings ScanWatch and the Biopac laboratory reference system. The plot displays aggregate data points derived from 28 participants evaluated across 4 distinct measurement conditions (seated rest trigger 1, seated rest trigger 2, supine respiration, and posttreadmill recovery). The solid red line represents the mean error, while the upper and lower solid blue lines define the 95% limits of agreement, which were calculated using a linear mixed-effects model to account for repeated measures.

**Figure 5.**
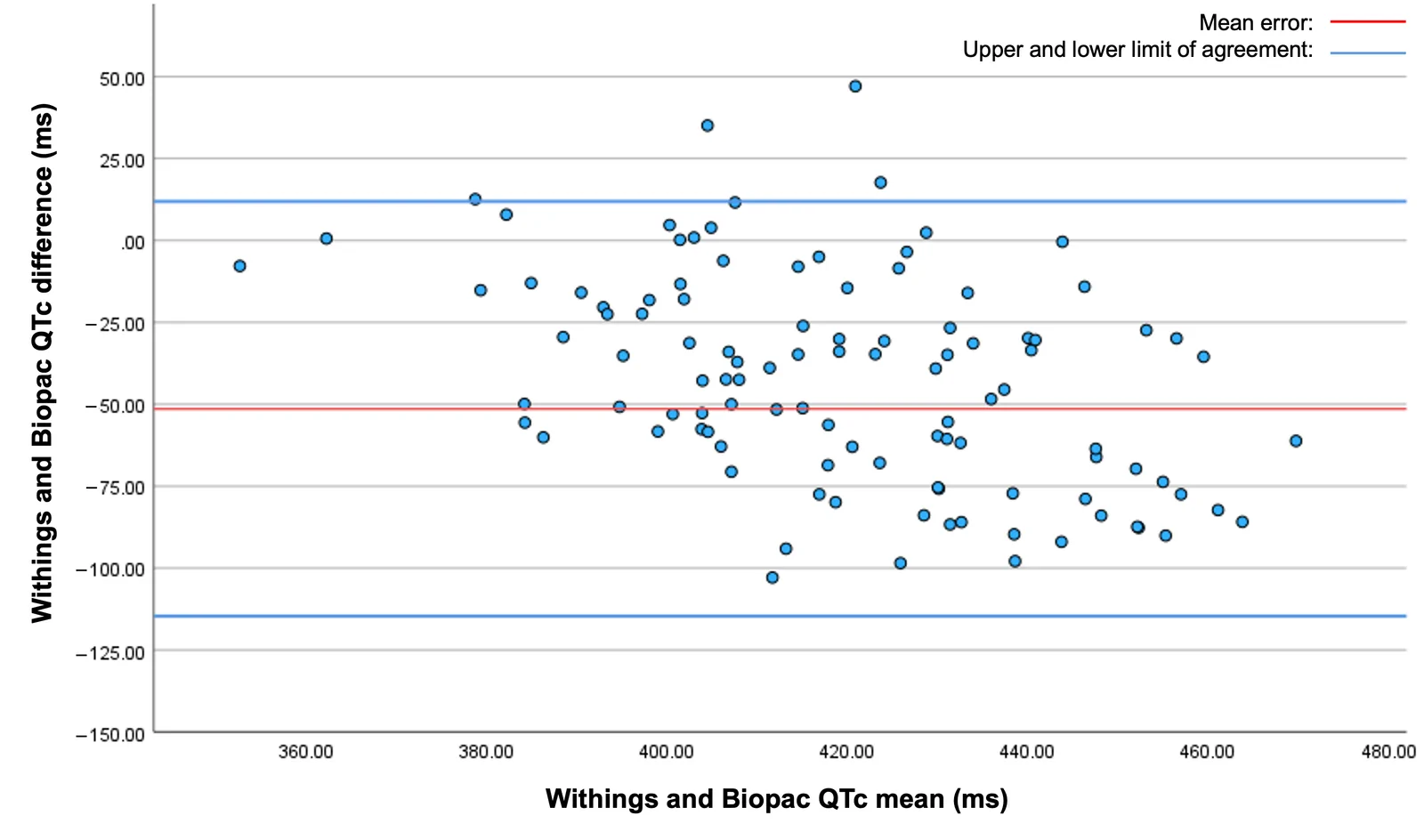
Bland-Altman plot illustrating the measurement agreement of corrected QT (QTc) intervals, calculated via the Bazett formula, between the Withings ScanWatch and the Biopac laboratory reference system. The plot displays aggregate data points derived from 28 participants evaluated across 4 distinct measurement conditions (seated rest trigger 1, seated rest trigger 2, supine respiration, and posttreadmill recovery). The solid red line represents the mean error, while the upper and lower solid blue lines define the 95% limits of agreement, which were calculated using a linear mixed-effects model to account for repeated measures.

Strong agreement was observed for RR intervals, supported by a strong *r*_rm_ coefficient (*r*_rm_=0.84; *P*<.001). The LMM confirmed that the fixed intercept for RR interval differences was not statistically significant (*P*=.36), with Bland-Altman analysis showing a slight ME of 9.4 milliseconds (Figure 3). Conversely, agreement was significantly weaker for both QT and QTc intervals, with *r*_rm_ coefficients indicating weak correlations (*r*_rm_=0.37; *P*<.05 and *r*_rm_=0.27; *P*<.05), respectively. The LMM fixed intercepts demonstrated that these mean discrepancies were statistically significant (*P*<.001 for both QT and QTc), revealing a systematic underestimation of −42.9 milliseconds for QT (Figure 4) and −51.4 milliseconds for QTc (Figure 5) by the Withings smartwatch (Table 2).

**Table 2.** Error, correlation, and Bland-Altman data for Withings ScanWatch compared with Biopac ECG.

Interval measured	Device error			Bland-Altman analysis		
	Error (ms), mean (SD)	MAPE^a^ (%; SD)	MAE^b^ (ms; SD)	Lower LoA^c^ (ms)	Upper LoA (ms)	Repeated-measures correlation (*r*_rm_)
RR	9.4 (52.3)	3.7 (0.9)	30.4 (6.7)	−93.1	111.9	0.84
QT	−42.9 (30.1)	9.5 (0.5)	40.3 (1.8)	−101.8	16.1	0.37
Corrected QT	−51.4 (32.3)	9.9 (1.0)	45.1 (5.3)	−114.7	11.9	0.27

a MAPE: mean absolute percent error.

b MAE: mean absolute error.

c LoA: limits of agreement.

### Withings ScanWatch Compared With Cardea SOLO

**Figure 6.**
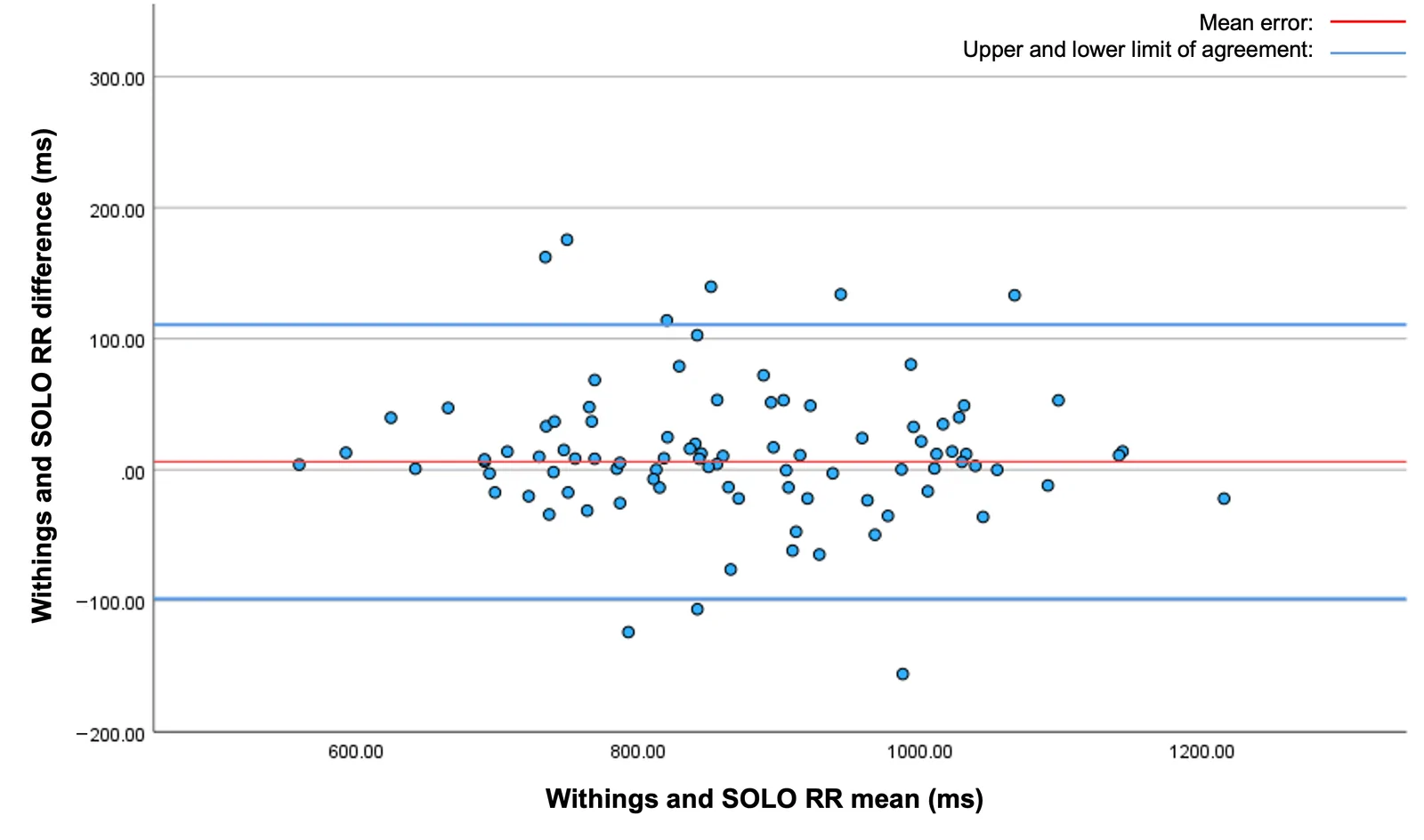
Bland-Altman plot illustrating the measurement agreement of beat-to-beat RR intervals between the Withings ScanWatch and the Cardea SOLO clinical ambulatory patch monitor. The plot displays aggregate data points derived from 28 participants evaluated across 4 distinct measurement conditions (seated rest trigger 1, seated rest trigger 2, supine respiration, and posttreadmill recovery). The solid red line represents the mean error, while the upper and lower solid blue lines define the 95% limits of agreement, which were calculated using a linear mixed-effects model to account for repeated measures.

**Figure 7.**
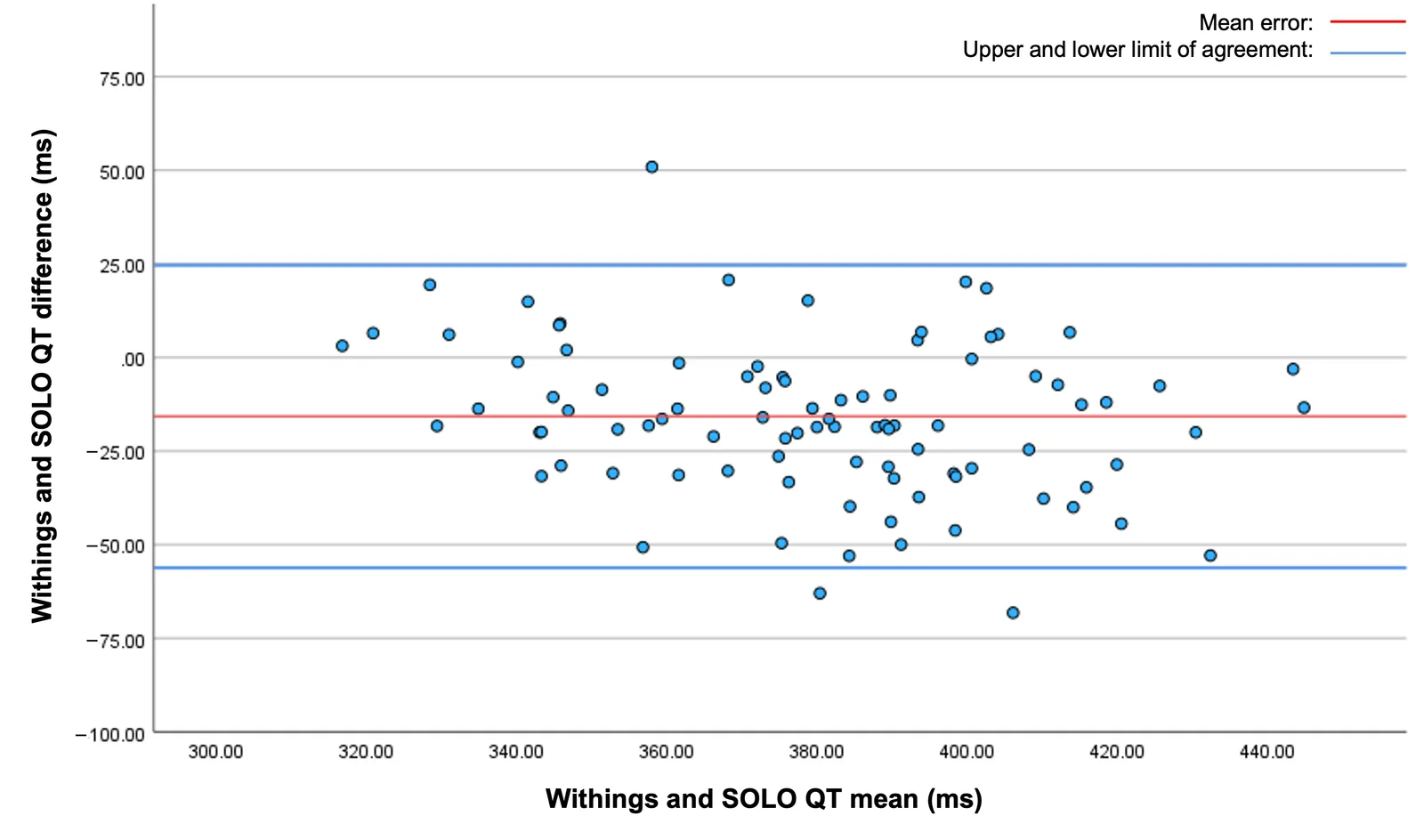
Bland-Altman plot illustrating the measurement agreement of manual QT intervals between the Withings ScanWatch and the Cardea SOLO clinical ambulatory patch monitor. The plot displays aggregate data points derived from 28 participants evaluated across 4 distinct measurement conditions (seated rest trigger 1, seated rest trigger 2, supine respiration, and posttreadmill recovery). The solid red line represents the mean error, while the upper and lower solid blue lines define the 95% limits of agreement, which were calculated using a linear mixed-effects model to account for repeated measures.

**Figure 8.**
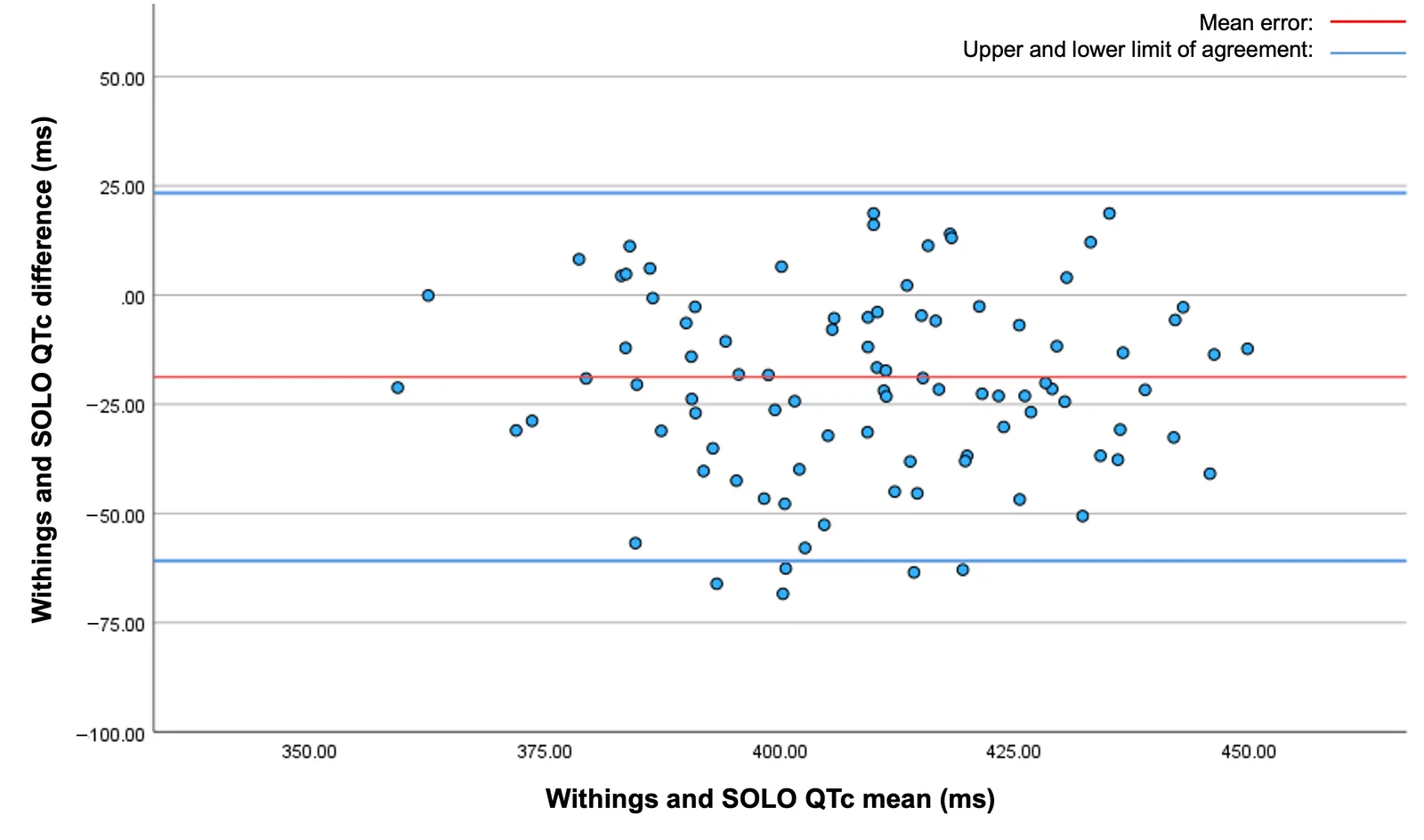
Bland-Altman plot illustrating the measurement agreement of corrected QT (QTc) intervals, calculated via the Bazett formula, between the Withings ScanWatch and the Cardea SOLO clinical ambulatory patch monitor. The plot displays aggregate data points derived from 28 participants evaluated across 4 distinct measurement conditions (seated rest trigger 1, seated rest trigger 2, supine respiration, and posttreadmill recovery). The solid red line represents the mean error, while the upper and lower solid blue lines define the 95% limits of agreement, which were calculated using a linear mixed-effects model to account for repeated measures.

Evaluation against the Cardea SOLO single-lead patch demonstrated a highly similar trend. The RR interval showed a strong correlative relationship (*r*_rm_=0.80; *P*<.001). The LMM indicated that the fixed intercept for RR interval differences was not statistically significant (*P*=.61), demonstrating strong clinical agreement with a low ME of 6.1 milliseconds (Figure 6). Agreement was notably lower for repolarization interval durations. The *r*_rm_ coefficient indicated a weak correlation for both the QT (*r*_rm_=0.37; *P*=.001) and QTc (*r*_rm_=0.27; *P*=.03) intervals. The LMM fixed intercepts demonstrated that these mean discrepancies were statistically significant (*P*<.001 for both QT and QTc), revealing a systematic underestimation of −15.8 milliseconds for QT (Figure 7) and −18.8 milliseconds for QTc (Figure 8) by the Withings smartwatch (Table 3). Consistent with the Biopac findings, Bland-Altman calculations highlighted a clear systematic pattern of interval underestimation by the wrist-worn consumer device.

**Table 3.** Error, correlation, and Bland-Altman data for Withings ScanWatch compared with Cardea SOLO.

Interval measured	Device error			Bland-Altman analysis		
	Error (ms), mean (SD)	MAPE^a^ (%; SD)	MAE^b^ (ms; SD)	Lower LoA^c^ (ms)	Upper LoA (ms)	Repeated-measures correlation (*r*_rm_)
RR	6.1 (53.4)	4.4 (0.6)	36.3 (5.6)	−98.5	110.7	0.80
QT	−15.8 (20.6)	5.4 (1.1)	21.3 (0.9)	−56.1	24.6	0.37
Corrected QT	−18.8 (21.5)	5.7 (1.6)	24.1 (0.5)	−60.8	23.3	0.27

a MAPE: mean absolute percent error.

b MAE: mean absolute error.

c LoA: limits of agreement.

## Discussion

### Principal Findings

This evaluation assessed the clinical agreement of intervals captured by a wrist-worn consumer smartwatch with those measured using both a continuous 3-lead laboratory ECG reference and an FDA-approved single-lead ambulatory patch monitor. Our primary findings demonstrated that while the wrist-worn device provided highly accurate, tightly correlated tracking of beat-to-beat RR intervals, it displayed pronounced systematic discrepancies when evaluating intracardiac microintervals. Specifically, the smartwatch consistently and uniformly underestimated both QT and QTc durations across all experimental testing conditions, demonstrating clinically meaningful absolute measurement errors. Crucially, the relatively wide Bland-Altman LoAs derived from our repeated-measures LMM underscored a substantial degree of individual-level variability, which further confirmed that the device could not be considered clinically interchangeable with standard diagnostic monitors at this time.

### Interpretation and Comparison With Prior Work

The strong correlative performance observed for the RR interval matches historical validation work verifying consumer smartwatches for ambient heart rate tracking and baseline rhythm surveillance. However, our data revealed that macrorhythm precision did not automatically equal accurate repolarization interval assessment. While previous studies exploring alternate consumer wearables reported tighter absolute interval alignment with clinical 12-lead ECG standards, the systematic underestimation found here limited this specific device’s immediate clinical interchangeability for acute arrhythmia screening.

This capability aligns well with the intended use of these devices for personal fitness tracking and general wellness monitoring [17]. Given the strong agreement at the individual level, our data suggested that smartwatches can be used to reliably track heart rate across multiple exercise intensity thresholds [23-25]. However, tracking capability has known boundaries; prior work indicates that consumer-grade device differences increase substantially at elevated heart rates (eg, heart rates ≥150 beats per min) [24], though participant heart rates in the current evaluation stayed within a window (≤130 beats per min) where this specific limitation was minimized.

Conversely, making informed clinical decisions regarding the management of conditions such as long QT syndrome requires a high level of both accuracy and precision because the normal range for repolarization intervals is exceptionally narrow [26,27]. Identifying true interval prolongation is crucial for guiding clinical management and pharmacotherapeutic choices, as it prevents the accidental administration of medications that could further extend the QT interval and precipitate life-threatening ventricular arrhythmias, including *torsade de pointes* [4,28].

This systematic error is fundamentally explained by distinct device architecture and lead geography constraints. Standard clinical practice relies on multilead arrays to actively identify the single vector capturing the maximum absolute QT duration [26,27], a diagnostic requirement that a single wrist-to-hand lead pathway cannot anatomically fulfill. Furthermore, while this evaluation focuses strictly on ventricular repolarization intervals (QT/QTc), consumer wearables face inherent diagnostic limitations regarding broader conduction disorders. For instance, detecting atrioventricular blocks relies entirely on the precise identification of the P wave and the subsequent PR interval. Because single-lead wrist-worn devices frequently suffer from baseline wandering and lower-amplitude signal-to-noise ratios, isolating distinct P-wave morphologies can be challenging, meaning the feasibility of QT tracking cannot be extrapolated to infer reliable atrioventricular block screening capabilities.

Certain structural limitations also extend to ambient signal processing. Consumer wearable electrode recordings are highly susceptible to early artifact noise and baseline wandering caused by initial skin-contact motion errors [29]. Selecting the single “clearest” tracing segment within a postexercise window can preferentially isolate periods of rapid heart rate deceleration during recovery. Because the Bazett formula is exceptionally sensitive to rapid heart rate fluctuations, this brief temporal mismatch relative to continuous tracking introduces substantial artifacts into calculated QTc metrics. Because the directional error is highly predictable and systematic, future software calibrations or corrective predictive algorithms could potentially offset this bias, transforming the device from an acute diagnostic screen into a highly valuable tool for passive, long-term intraindividual trend tracking.

### Limitations

Several critical limitations qualify these findings. First, the evaluation sample size (N=28) was limited and established as a convenience sample for a pilot study. While this cohort size provided sufficient statistical power to detect robust, high-amplitude physiological trends, such as the strong beat-to-beat agreement observed across RR intervals, it restricted our overall statistical power when assessing highly variable microintervals such as the QT and QTc segments. Consequently, our findings may have limited generalizability to broader clinical populations. Furthermore, because the study comprised healthy, younger adults free from underlying cardiac disease states, the device was not evaluated in a true pathological cohort exhibiting baseline repolarization abnormalities or manifest conduction defects. Therefore, its performance in true high-risk clinical populations remains unverified. Second, data across the distinct physiological testing environments were aggregated for the primary repeated-measures statistical analyses. While using LMMs allowed us to account for within-participant clustering, pooling data across conditions may have inadvertently masked subtle accuracy variations caused by active postural changes or ambient motion artifact. Crucially, because all postexercise smartwatch triggers were executed while participants were seated quietly at rest to ensure tracing viability, this protocol did not capture the severe motion and muscle-activation artifacts associated with active, ambulatory movement. Finally, all manual interval calipers were placed by a single independent investigator. Although standardized operating procedures were strictly enforced, microinterval end point determination remains inherently operator dependent.

### Public Health Implications

While the evaluated smartwatch serves as a convenient and precise method for monitoring heart rate regularity and tracking long-term trends, its systematic underestimation of QT and QTc intervals makes it currently unreliable as a standalone clinical tool for acute arrhythmia risk stratification. In a broader public health context, introducing uncalibrated consumer-facing interval metrics can trigger significant unintended health care use consequences. Minor, benign interval variations reported as abnormal by a device can generate unwarranted patient anxiety, increase psychological stress, and drive an influx of false-positive emergency or specialist consultations, imposing a substantial cost and burden on health care systems without definitively improving clinical outcomes [30]. Future iterations of consumer ECG technologies must prioritize lead-specific algorithmic calibration to safely unlock the vast potential of accessible, population-level cardiac tracking.

### Conclusions

In summary, the Withings ScanWatch offers a convenient and reasonably accurate method for tracking heart rate and detecting basic rhythm abnormalities. However, its current limitations in accurately measuring QT and QTc intervals render it unreliable for screening for many of the most common and deadly arrhythmias. As wearable technology continues to advance, future iterations of these devices may address these limitations, paving the way for more comprehensive and clinically valuable cardiac monitoring tools.

## Abbreviations

AF: atrial fibrillation
ECG: electrocardiogram
FDA: Food and Drug Administration
LMM: linear mixed-effects model
LoA: limits of agreement
MAE: mean absolute error
MAPE: mean absolute percent error
ME: mean error
MET: metabolic equivalent of task
QTc: corrected QT interval
